# Role of C/EBP-β in Methamphetamine-Mediated Microglial Apoptosis

**DOI:** 10.3389/fncel.2019.00366

**Published:** 2019-08-21

**Authors:** Xuebing Chen, Jiancong Lu, Xu Zhao, Chuanxiang Chen, Dongfang Qiao, Huijun Wang, Xia Yue

**Affiliations:** School of Forensic Medicine, Southern Medical University, Guangzhou, China

**Keywords:** methamphetamine, C/EBP-β, lipocalin2, microglia, apoptosis

## Abstract

Methamphetamine (MA) is a widely abused psychoactive drug that primarily damages the nervous system. However, the involvement of MA in the survival of microglia remains poorly understood. CCAAT-enhancer binding protein (C/EBP-β) is a transcription factor and an important regulator of cell apoptosis. Lipocalin2 (lcn2) is a known apoptosis inducer and is involved in many cell death processes. We hypothesized that C/EBP-β is involved in MA-induced lcn2-mediated microglial apoptosis. To test this hypothesis, we measured the protein expression of C/EBP-β after MA treatment and evaluated the effects of silencing C/EBP-β or lcn2 on MA-induced apoptosis in BV-2 cells and the mouse striatum after intrastriatal MA injection. MA exposure increased the expression of C/EBP-β and stimulated the lcn2-mediated modulation of apoptosis. Moreover, silencing the C/EBP-β-dependent lcn2 upregulation reversed the MA-induced microglial apoptosis. The *in vivo* relevance of these findings was confirmed in mouse models, which demonstrated that the microinjection of anti-C/EBP-β into the striatum ameliorated the MA-induced decrease survival of microglia. These findings provide a new insight regarding the specific contributions of C/EBP-β-lcn2 to microglial survival in the context of MA abuse.

## HIGHLIGHTS

- Methamphetamine upregulated the expression of C/EBP-β in microglia.
- Methamphetamine increased the expression of lcn2 through C/EBP-β pathway.
- C/EBP-β is involved in Methamphetamine-induced lcn2-mediated microglial apoptosis.

## Introduction

Methamphetamine (MA), a widely abused psychoactive drug, has long been known to elevate the concentration of cytoplasmic dopamine through binding with the dopamine transporter and to ultimately induce neuronal death (Gouzoulis-Mayfrank et al., 2017; Li et al., 2017; Moszczynska and Callan, 2017). Microglial activation that is thought to participate in either protoxic or protective mechanisms in the MA-induced neurotoxicity (Coelho-Santos et al., 2012; Fernandes et al., 2016). Microglia, the resident macrophages of the central nervous system (CNS), play an important role in immune surveillance, and compromised microglial function is associated with various neurological pathologies (Streit et al., 1999). Accumulating evidence suggests that activated microglia have been associated with MA-induced neurotoxicity both *in vitro* and *in vivo* (Urrutia et al., 2013; Goncalves et al., 2017; Shaerzadeh et al., 2018). In addition, chronic MA abuse is also associated with microglial activation in the brains of MA-addicted humans (Sekine et al., 2008). Activated microglia eventually undergo apoptosis by a process known as activation-induced cell death (AICD) through the regulation of apoptotic proteins, including caspases and the BCL2 family of proteins (Zhang et al., 2016). Although MA is known to induce microglial activation, whether MA induces AICD in microglia and the molecular mechanisms in this process remain elusive.

CCAAT-enhancer binding protein (C/EBP-β) is a member of the C/EBP family of bZIP transcription factors that bind DNA as dimers. Through the alternative use of three translation initiation codons, three C/EBP isoforms (called LAP^∗^, LAP, and LIP) are translated from a single mRNA (Bradley et al., 2003). LAP^∗^ and LAP function as transcriptional activators, while the LIP function as a transcriptional repressor in a dominant negative fashion because of lacking a transactivation domain. Recent studies have reported that C/EBP-β participates in multiple cellular functions, such as cell death and survival (Zahnow, 2009; van der Krieken et al., 2015; Xu X. et al., 2018). Many studies have demonstrated that C/EBP-β is an important regulator of the pro-inflammatory program in microglia (Strohmeyer et al., 2014). Our recent study demonstrated that C/EBP-β mediated neuronal apoptosis through mitochondrial pathways (Xu X. et al., 2018). MA can induce microglial apoptosis (Zhang et al., 2016; Sharikova et al., 2018; Du et al., 2019). However, whether C/EBP-β is involved in MA-induced microglial apoptosis remains elusive.

Lipocalin2 (lcn2) is involved in cell death in different situations (Bi et al., 2013; Kim et al., 2016). The proapoptotic role of lcn2 is thought to be involved in mitochondrial pathways (Jin et al., 2011; Chien et al., 2012). The expression of lcn2 is regulated by a network of stimuli acting through different transduction pathways. Notably, C/EBPs are implicated in the transcriptional control of lcn2 (Jha et al., 2015). C/EBPs have transcription factor binding sites that influence lcn2 promoter activity (Cowland et al., 2003; Shen et al., 2006). The LPS-induced upregulation of C/EBP-β increased lcn2 expression in the lung and liver (Sunil et al., 2007). We hypothesized that C/EBP-β regulates lcn2 expression after MA exposure. In the current work, we investigated the role of C/EBP-β in MA-induced microglial death.

## Materials and Methods

### Animals and Tissue Collection

Male C57BL/6 mice (6–8 weeks old) were purchased from the Laboratory Animal Center of Southern Medical University (SMU), Guangzhou, China. Each mouse was housed singly at room temperature with a 12 h light/12 h dark cycle. Food and water were available *ad libitum*. Animal care and experimental procedures were approved by the Institutional Animal Care and Use Committee at SMU and followed the latest NIH Guidelines for the Care and Use of Laboratory Animals (National Research Council, 2011). The animals were habituated to the animal facility for 1 week before the experiments began. The mice were divided randomly into control group, acute group and subacute group experimental groups (*n* = 5–8/group). The acute group received two injections [15 mg/kg, intraperitoneal (i.p.), at 12-h intervals]. The subacute group received eight injections [15 mg/kg, intraperitoneal (i.p.), at 12-h intervals] of either saline vehicle or MA (>99% purity; National Institutes for Food and Drug Control, Guangzhou, China). This exposure paradigm was based on previous studies (Winek et al., 2001; Cadet et al., 2003; Krasnova and Cadet, 2009; Zhang et al., 2016). The animals were euthanized 48 h after the last injection. Brain samples were quickly removed, and the striatal tissues were dissected on ice and stored at −80°C until use.

### Cell Culture and MA Treatments

The mouse microglia cell line (BV-2) was purchased from the Shanghai Cell Bank of the Chinese Academy of Sciences. BV-2 cells were cultured in high glucose DMEM (Gibco, CA, United States) supplemented with 10% fetal bovine serum (FBS) in a humidified incubator (37°C, 5% CO_2_, 95% air). BV-2 cells were pretreated with a range of MA concentrations (0.25–1.5 mM) for 24 h to evaluate the corresponding apoptotic events. This concentration was chosen for subsequent experiments based on previous studies (Frank et al., 2016; Zhang et al., 2016).

### Western Blot Analysis

BV-2 cells and the brain tissue from mice exposed to vehicle or MA were lysed in ice-cold RIPA buffer with protease inhibitor. Protein concentrations were determined with the BCA-100 Protein Quantitative Analysis Kit (Biocolors, Shanghai, China). Protein samples were separated by 10–15% sodium dodecyl sulfate polyacrylamide gel electrophoresis (SDS–PAGE) and transferred onto 0.22 mm polyvinylidene difluoride (PVDF) membranes (Millipore, Billerica, MA, United States). The membranes were blocked at room temperature for 2 h in 5% non-fat milk or for 1 h in 5% bovine serum albumin (BSA) (Biosharp, Guangzhou, China) and then incubated with specific primary antibodies overnight at 4°C, followed by incubation with horseradish peroxidase conjugated secondary antibodies for 1 h at room temperature. The primary antibodies were as follows: anti C/EBP-β (1:1000) and anti-lcn2 (1:500) from Abcam (Cambridge, United Kingdom) and anti-cleaved caspase3 (1:1000), anti-cleaved PARP (1:1000), anti-bax (1:1000), anti-bcl2 (1:1000), anti-cytochrome c (cyto-c, 1:1000), and anti-β-actin (1:1000) purchased from the Cell Signaling Technology (Danvers, MA, United States). The protein bands were developed with Super ECL Western Blotting Detection Reagents. The expression of the housekeeping gene β-actin was used as a reference control. The signal of band intensities was quantitated by a Gel-Pro analyzer (Media Cybernetics, Inc., Rockville, MD, United States).

### siRNA and Transient Transfection

Specific small interfering RNA (siRNA) sequences targeting C/EBP-β and lcn2 were synthesized by GenePharma (Shanghai, China) as follows: C/EBP-β siRNA: 5′-GCGACAAGAAGCTGACTTT-3′; and lcn2 siRNA: 5′-CCAAGCTGCCAGACTATAA-3′. The scrambled siRNA sequence was used as a control. siRNAs were dissolved in diethylpyrocarbonate (DEPC) water at a concentration of 20 μM. Cells were placed on a 6-well plate at a density of 5 × 10^5^ cells/well. When cells reached 60% confluence, 20 μmol siRNA, and 5 μl of Lipofectamine 3000 Reagent (Invitrogen, Carlsbad, CA, United States) were added to Opti-MEM medium (Gibco BRL, Paisley, United Kingdom). The mixed solution was incubated for 15 min at room temperature. Then, cells were incubated with standard culture medium for 12–18 h and thereafter were exposed to 1.0 mM MA in non-serum medium for another 24 h.

### Immunofluorescence Labeling

BV-2 cells were seeded in confocal dishes with different treatments, and brain tissues were fixed with paraformaldehyde and permeabilized with 0.1% Triton-X. After blocking with 5% BSA, cells and tissue sections were incubated with rabbit C/EBP-β (1:500; Abcam) and lcn2 (1:100; Abcam) at 4°C. After washing and incubating with Alexa 488-conjugated mouse anti-rabbit IgG (1:500; Invitrogen), Alexa 555-conjugated goat anti-rabbit IgG (1:500; Invitrogen), and Alexa 594-conjugated goat anti-rabbit IgG (1:500; Invitrogen), the cells were stained with 4′,6′-diamidino-2-phenylindole (DAPI). The secondary antibodies were purchased from the Thermo Life Technologies (CA, United States). The images were observed by confocal microscopy (Carl ZeissLSM 880 with Airyscan, Germany) using the same settings to improve the contrast.

### TUNEL Staining

BV-2 cells were seeded on confocal dishes overnight. Cells were pretreated with lipo/scrambled siRNA and then exposed to MA (1.0 mM) or vehicle for 24 h. Visualization of DNA fragmentation was performed using the fluorometric terminal deoxynucleotidyl transferase (TdT) dUTP nick-end labeling (TUNEL) system for apoptotic cells (KeyGen Biotech, China) according to the manufacturer’s instructions. Both TUNEL- and DAPI-positive cells were counted by Image J software. Data are calculated based on the total number of TUNEL-positive cells.

### Annexin V Apoptosis Staining

BV-2 cells were placed on a 6-well plate at a density of 3–5 × 10^5^cells/ml. Cells were pretreated with lipo/scrambled siRNA and then exposed to MA (1.0 mM) or vehicle for 24 h. The cells were collected after washing twice with 4°C PBS and stained with Annexin V-FITC and propidium iodide (PI; 4A Biotech Co., Ltd., Nanjing, China) according to instructions of the Annexin V Apoptosis Detection Kit. The percentage of apoptotic cells was quantified with a flow cytometry system (FACSCalibur; BD Biosciences, Franklin Lakes, NJ, United States). Either early apoptosis (Annexin V-FITC-positive, PI-negative) or late apoptosis (Annexin V-FITC-positive, PI-positive) was considered apoptotic cells.

### Measurement of the Mitochondrial Membrane Potential

The fluorescent dye JC-1 (KeyGen Biotech) was used to assess the change in mitochondrial membrane potential. Briefly, cells seeded into confocal dishes were incubated with a mixture of 1 μl of JC-1 staining fluid and 500 μl of incubation buffer in the dark at 37°C for 30 min, washed twice with the incubation buffer, and observed by confocal microscopy (Carl ZeissLSM 880 with Airyscan, Germany).

### shRNA and Stereotaxic Injection

The shRNA synthesis and stereotaxic injection protocol were based on our recent studies (Huang et al., 2015; Li et al., 2017). In brief, the C/EBP-β shRNA sequence (5′-TTTAAGTGATTACTCAGGG-3′) was cloned into the pGC-LV vector and transfected into human embryonic kidney (HEK 293FT) cells with the pHelper 1.0 and 2.0 vectors. The LV-shC/EBP-β lentivirus was harvested (10^9^ transducing units/ml). LV-GFP was used as a control virus. The C57 mice were randomly divided into four groups (*n* = 5–8/group): the LV-GFP group, LV-shC/EBP-β group, LV-GFP + MA group, and LV-shC/EBP-β + MA group. LV-shC/EBP-β lentivirus or LV-GFP lentivirus were bilaterally injected into the striatum [coordinates relative to bregma: anteroposterior (AP) +0.4 mm; mediolateral (ML) ±2 mm; and dorsoventral (DV) −3.5 mm] via a stereotaxic apparatus (Yuan et al., 2018). After 4 days, the animals were injected with saline vehicle or MA (15 mg/kg × 8 injections, i.p., at 12-h intervals) and were euthanized 48 h after the final injection. Brain samples were quickly removed and stored at −80°C until use.

### Statistical Analysis

All data were expressed as the mean ± standard deviation (SD) with at least three independent replicates. Statistical analysis was performed using one-way ANOVA followed by least significant difference (LSD) *post hoc* analysis and independent-samples *t*-test (as appropriate) with the statistical software SPSS version 20.0 (SPSS Inc., Chicago, IL, United States). *P* < 0.05 was considered statistically significant.

## Results

### MA Exposure Increased C/EBP-β Isoform LAP Expression *in vivo* and *in vitro*

**FIGURE 1:**
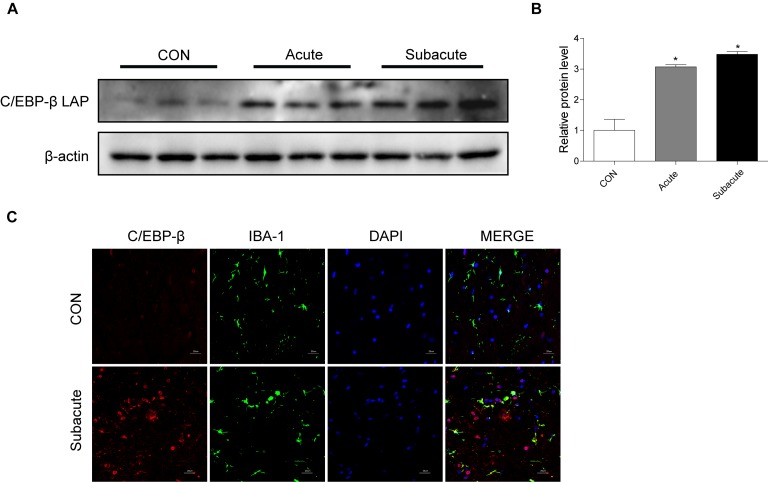
MA exposure increases C/EBP-β isoform LAP expression *in vivo*. Representative Western blots **(A)** and quantitative analyses **(B)** of C/EBP-β in the corpus striatum tissues of mice from the control group, acute group and subacute group. *N* = 5–8 animals/group. Fold induction relative to the vehicle-treated group (^∗^*p* < 0.05 vs. the vehicle-treated group using one-way ANOVA).β-actin was used as a loading control. All data are presented as the mean ± SD. **(C)** Representative immunofluorescence microscopy images of C/EBP-β (red) in the striatal microglia (green, IBA-1) of mice in the vehicle-treated group, subacute MA-treated group (scale bar = 20 μm). Nuclei were counterstained with DAPI (blue). C/EBP-β (red) merged with DAPI (blue) was pink. All data were expressed at least three independent replicates.

**FIGURE 2:**
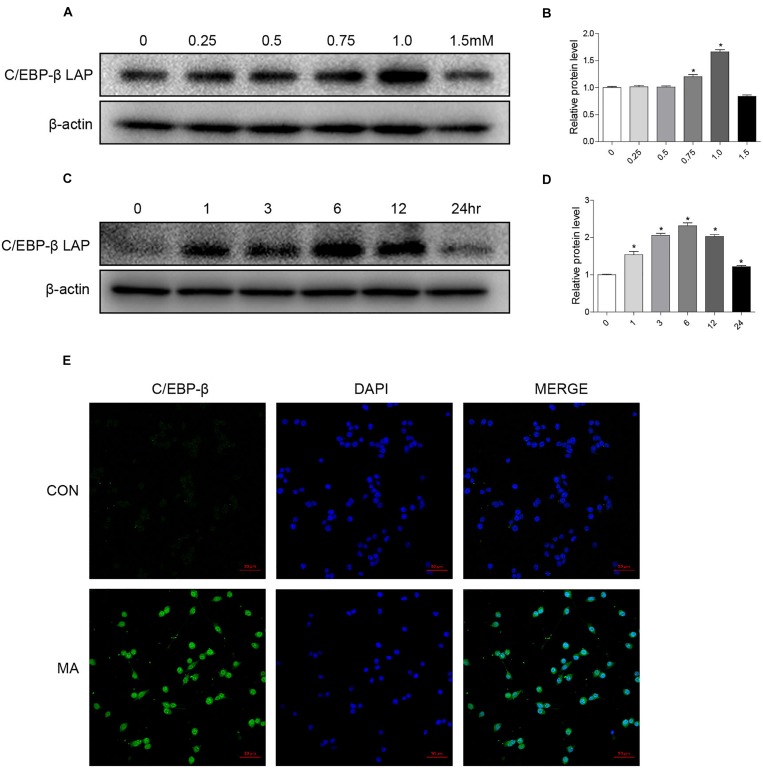
MA exposure increases C/EBP-β isoform LAP expression *in vitro*. Microglial BV-2 cells were treated with 0.25–1.5 mM MA for 24 h **(A,B)** and exposed to 1.0 mM MA for 1–24 h **(C,D)**. Representative immunofluorescence microscopy images of C/EBP-β (green) in BV-2 cells in the vehicle-treated group and MA-treated group (**E**, scale bar = 50 μm). Nuclei were counterstained with DAPI (blue). All data were expressed at least three independent replicates.β-actin was used as a loading control. The fold induction relative to that in the vehicle-treated group is shown. All data are presented as the mean ± SD, ^∗^*p* < 0.05 vs. the vehicle-treated group using one-way ANOVA.

To address the role of the C/EBP-β isoform LAP after MA exposure, C57BL/6 mice were treated with MA or a control, and the expression of the C/EBP-β isoform LAP was detected. Western blot analysis showed that the expression of the C/EBP-β isoform LAP in the striatum of MA-treated mice was significantly increased in both the acute group and subacute group, and this change was at a greater extent after longer exposure (by 3.48-fold for subacute group and 3.07-fold for acute group, Figures 1A,B). MA-treated mice was significantly increased in the acute group while decreased in the subacute group (Supplementary Figures 1A,B). Therefore, the subacute group was selected for further studies. Immunofluorescence staining for the striatum (Figure 1C) showed C/EBP-β was significantly increased in the subacute group. Furthermore, BV-2 cells were treated with different concentrations of MA (0, 0.25, 0.5, 0.75, and 1.5 mM) for different durations (0, 1, 3, 6, 12, or 24 h), and C/EBP-β was determined by Western blot analysis. The results showed that the expression levels of the C/EBP-β isoform LAP, but not C/EBP-β LIP (data not shown), increased significantly in a dose- and time-dependent manner in MA-treated BV-2 cells (Figures 2A–D). The results from the immunofluorescence staining of BV-2 cells (Figure 2E) showed that MA exposure increased the expression of C/EBP-β. Collectively, these data suggested that MA exposure induces the expression of C/EBP-β in microglia both *in vitro* and *in vivo*.

### MA Increased Apoptosis in the BV-2 Cells

**FIGURE 3:**
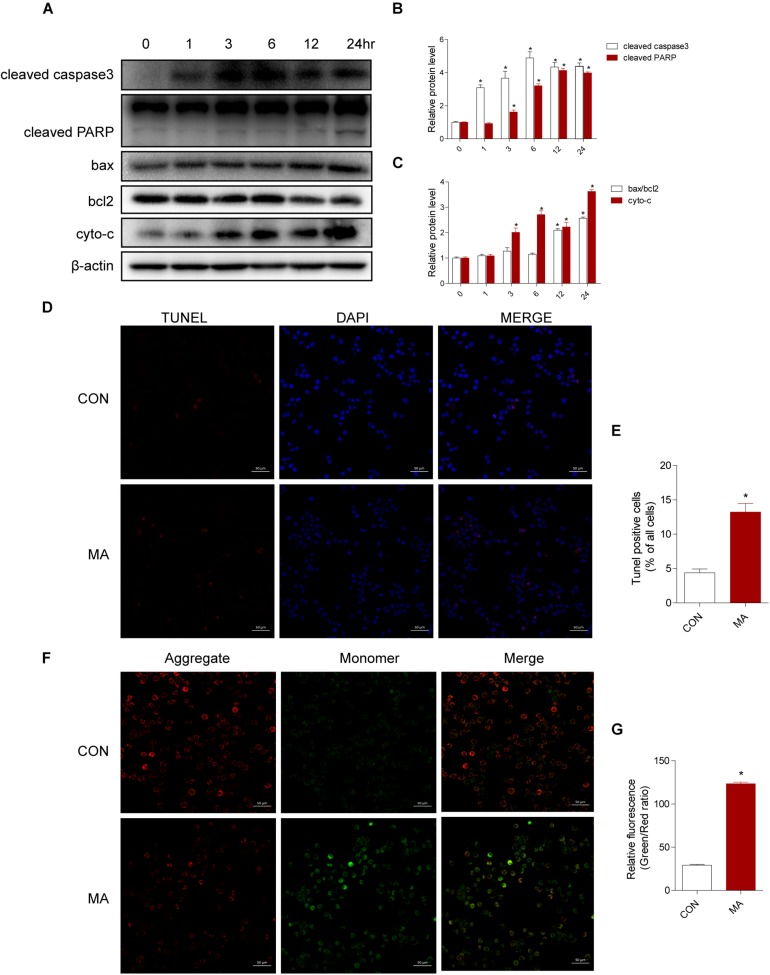
MA increases apoptosis *in vitro*. Microglial BV-2 cells were treated with 1.0 mM MA for 1–24 h **(A–C)**. The protein expression levels of cleaved caspase3 and cleaved PARP, the ratio of bax/bcl2 and cytochrome c were determined with Western blot and quantitative analyses. Representative data from three independent experiments are shown. β-actin was used as a loading control. Apoptotic cells were subjected to TUNEL staining (**D**, scale bar = 50 μm). Nuclei were counterstained with DAPI (blue). Quantitative analysis of the percentage of apoptotic cells using a standard cell counting method with the TUNEL assay **(E)**. Confocal microscopy analysis using JC-1 staining revealed that MA (1.0 mM, 24 h) challenge decreased the MΨm level in BV-2 cells (**F**, scale bar = 50 μm). Red stands for the JC-1 aggregate fluorescence from healthy mitochondria, and green exhibits cytosolic JC-1 monomers. The increased ratio of JC-1 monomers (green) to JC-1 aggregates (red) stands for the decreased MΨm. The fold induction relative to that in vehicle-treated cells is shown **(G)**. Data are presented as the means ± SD with at least three independent replicates. ^∗^*p* < 0.05 vs. the saline vehicle-treated control group. Data in panels **(B,C)** were analyzed by one-way ANOVA. Data in panels **(E,G)** were analyzed by Student’s *t*-test.

To assess the effect of MA exposure in microglial cell toxicity, BV-2 cells were treated with 0.25–1.5 mM MA for 24 h and 1.0 mM MA for 1–24 h. Then, Western blot analysis was performed to measure cleaved caspase3, cleaved PARP, the ratio of bax/bcl-2, and cyto-c expression levels, which were also significantly increased in a dose-dependent manner after MA exposure (Supplementary Figures 2A–C). As shown in Figures 3A–C, the apoptosis-related proteins were significantly increased in a time-dependent manner after MA exposure. This result was further confirmed by TUNEL staining (Figures 3D,E). MA treatment resulted in increasing the ratio of JC-1 monomers (green) to JC-1 aggregates (red), standing for the loss of mitochondrial membrane potential (MΨm) in BV-2 cells (Figures 3F,G). Together, these results indicated that the accumulation of MA might play a key role in microglial cell toxicity.

### The C/EBP-β Isoform LAP Is Responsible for the MA-Induced Apoptosis in BV-2 Cells

**FIGURE 4:**
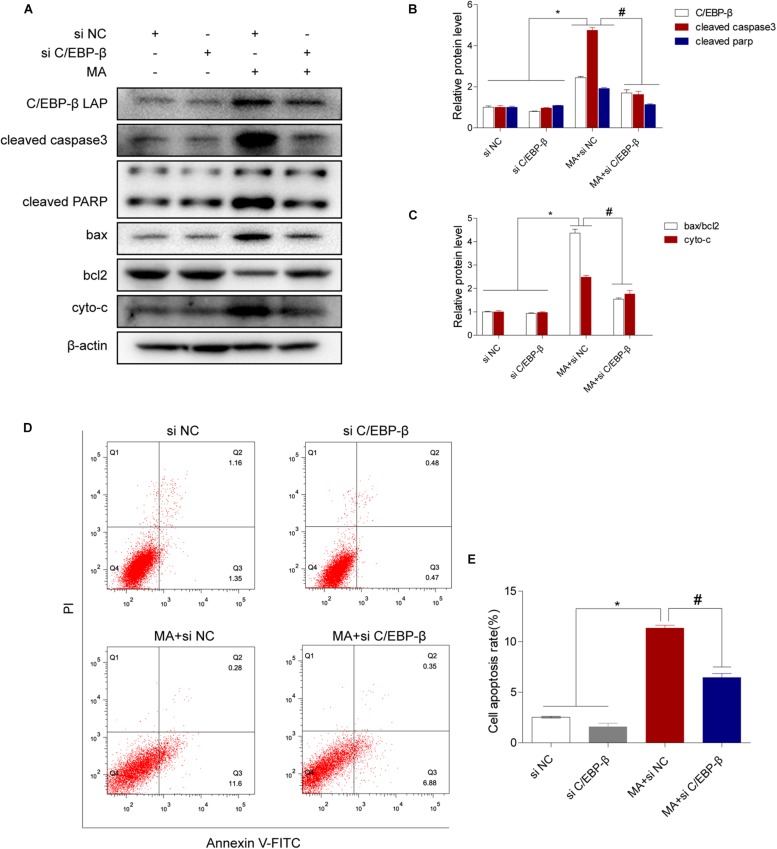
The C/EBP-β isoform LAP is responsible for MA-induced apoptosis *in vitro*. Representative Western blots **(A)** and quantitative analyses **(B,C)** of C/EBP-β, cleaved caspase3 and cleaved PARP, the ratio of bax/bcl2 and cytochrome c in BV-2 cells exposed to vehicle + control siRNA, 1.0 mM MA + control siRNA, vehicle + C/EBP-β siRNA and 1.0 mM MA + C/EBP-β siRNA. All data were expressed at least three independent replicates. β-actin was used as a loading control. Apoptotic cells were stained and analyzed with flow cytometry **(D)**. The percentage of apoptosis is presented as the mean ± SD **(E)**. ^∗^*p* < 0.05 vs. the saline vehicle-treated control group, ^#^*p* < 0.05 vs. the scrambled + MA group (one-way ANOVA).

**FIGURE 5:**
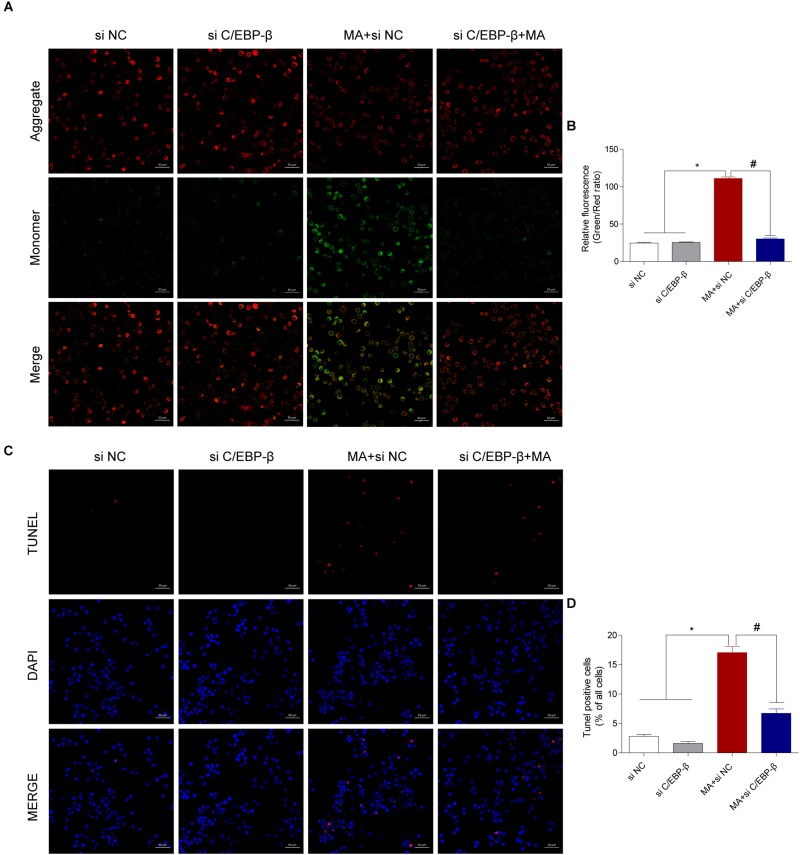
Silencing C/EBP-β decreases the cell apoptosis induced by MA exposure *in vitro* with the TUNEL assay and JC-1 staining. Representative images of the JC-1 staining (**A**, scale bar = 50 μm) and TUNEL assay (**C**, scale bar = 50 μm) in BV-2cells from different groups: vehicle + control siRNA, 1.0 mM MA + control siRNA, vehicle + C/EBP-β siRNA, and 1.0 mM MA + C/EBP-β siRNA for 24 h exposure. Bar graphs show the ratio of aggregated and monomeric JC-1 **(B)**. The percentage of apoptosis is presented as the mean ± SD **(D)**. ^∗^*p* < 0.05 vs. the saline vehicle-treated control group, ^#^*p* < 0.05 vs. the scrambled + MA group (one-way ANOVA).

We next evaluated the involvement of C/EBP-β in the MA-induced activation of the intrinsic apoptosis pathway. We used siRNAs targeting C/EBP-β to silence its expression and then examined the effects on MA-induced apoptosis in BV-2 cells. Western blot analysis showed that the MA-induced increases in apoptotic-related proteins were significantly blocked by siRNAs targeting C/EBP-β (Figures 4A–C). This result was further confirmed by flow cytometry (Figures 4D,E), the JC-1 assay (Figures 5A,B) and the TUNEL assay (Figures 5C,D). These results suggest that knockdown of C/EBP-β can effectively decrease MA-induced apoptosis in microglia.

### Lcn2 Is a Downstream Target of C/EBP-β

**FIGURE 6:**
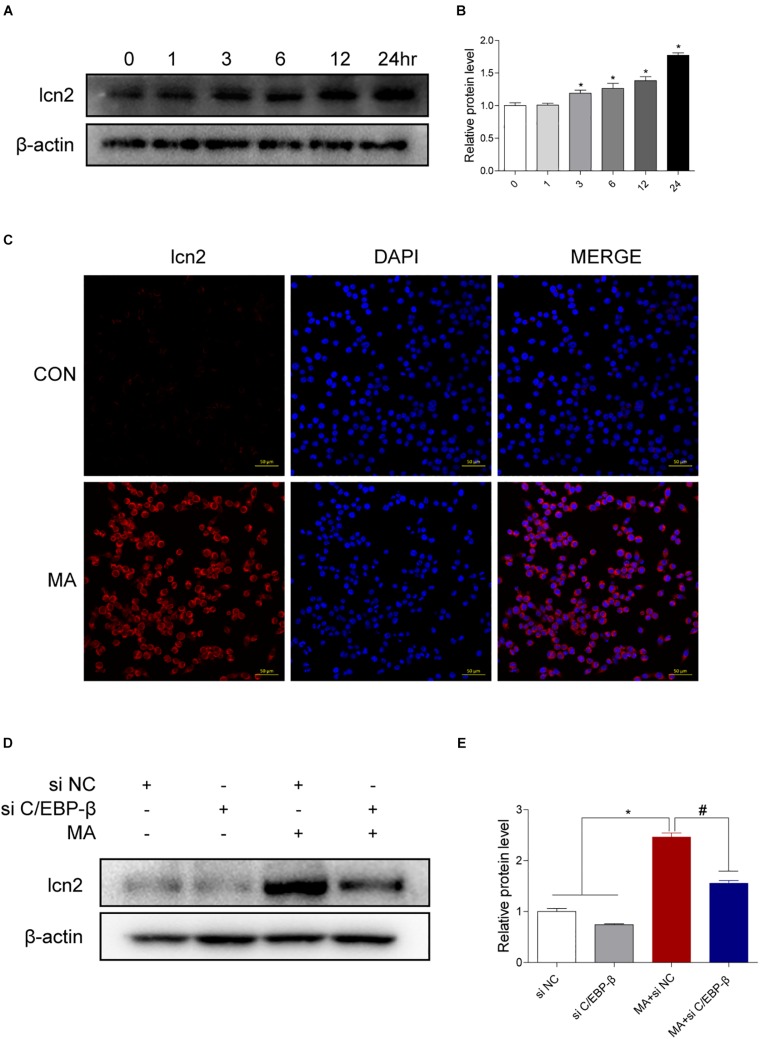
Lcn2 is a downstream target of C/EBP-β. **(A,B)** Representative Western blots of lcn2 expression in BV-2 cells exposed to MA (0–24 h). **(C)** Representative immunofluorescence microscopy images of lcn2 (red) in BV-2 cells in the vehicle-treated group and MA-treated group (scale bar = 50 μm). Nuclei were counterstained with DAPI (blue). Western blot results revealed that the MA-induced lcn2 increased expression could be blocked by preincubating with C/EBP-β siRNA **(D,E)**. β-actin was used as a loading control. The fold induction relative to that in vehicle-treated cells is shown. Data are presented as the means ± SD. ^∗^*p* < 0.05 vs. the saline vehicle-treated control group, ^#^*p* < 0.05 vs. the scrambled + MA group (one-way ANOVA). All data were expressed at least three independent replicates.

Therefore, we hypothesized that C/EBP-β may mediate MA-induced microglial apoptosis via upregulation of lcn2 expression. To investigate whether lcn2 is the target of C/EBP-β, Western blot and immunofluorescence staining were used, and the results showed that lcn2 was increased after MA exposure (Figures 6A–C). In addition, we observed that the MA-induced increases in lcn2 expression were significantly blocked after C/EBP-β knockdown in 1.0 mM MA-exposed BV-2 cells (Figures 6D,E), suggesting that lcn2 may be a downstream protein of C/EBP-β.

### Lcn2 Plays a Critical Role in C/EBP-β-Mediated MA-Induced Apoptotic Signaling Pathways

**FIGURE 7:**
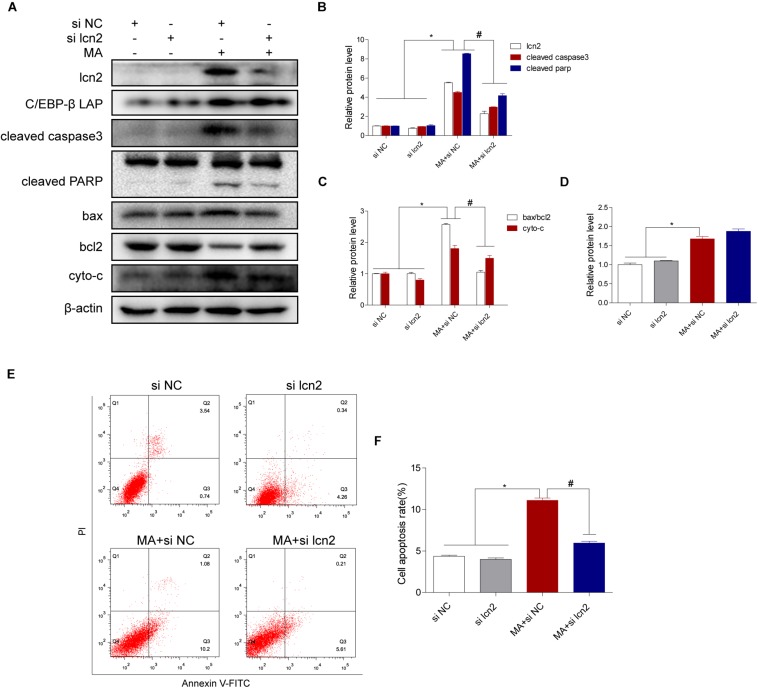
Lcn2 plays a critical role in C/EBP-β-mediated MA-induced apoptotic signaling pathways. Representative Western blots of lcn2, cleaved caspase3 and cleaved PARP, the ratio of bax/bcl2, cytochrome c (**A–C**, respectively) and C/EBP-β **(D)** in BV-2 cells exposed to vehicle + control siRNA, 1.0 mM METH + control siRNA, vehicle + lcn2 siRNA, and 1.0 mM METH + lcn2 siRNA. All data were expressed at least three independent replicates. β-actin was used as a loading control. Apoptotic cells were stained and analyzed with flow cytometry **(E)**. The percentage of apoptosis is presented as the mean ± SD **(F)**. ^∗^*p* < 0.05 vs. the saline vehicle-treated control group, ^#^*p* < 0.05 vs. the scrambled + MA group (one-way ANOVA).

**FIGURE 8:**
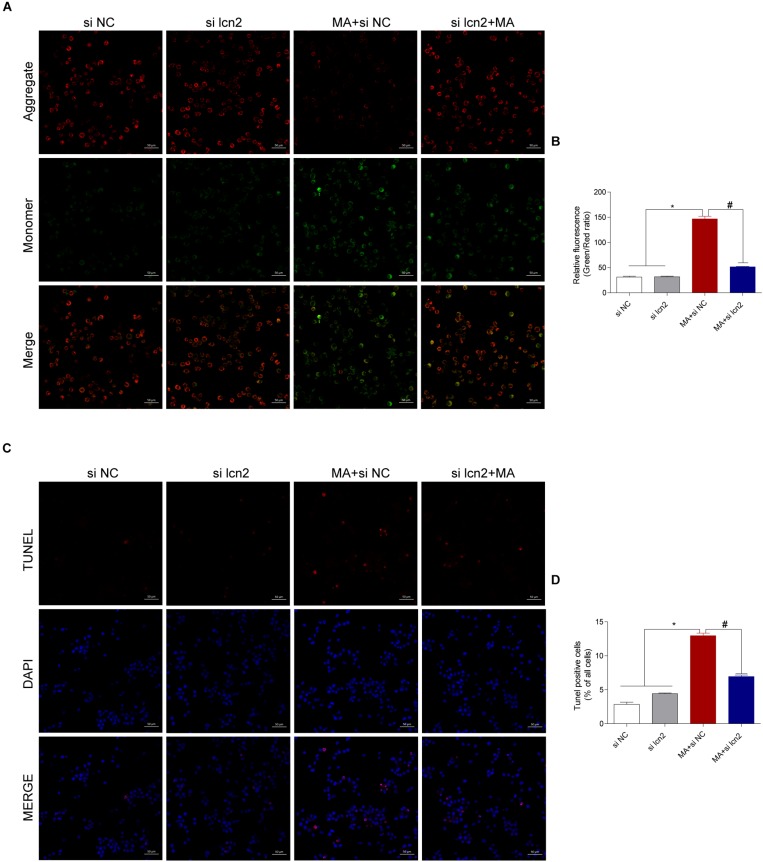
Silencing lcn2 decreases the cell apoptosis induced by MA exposure *in vitro* with the TUNEL assay and JC-1 staining. Representative images of the JC-1 staining (**A**, scale bar = 50 μm) and TUNEL assay (**C**, scale bar = 50 μm) in BV-2 cells from different groups: vehicle + control siRNA, 1.0 mM MA + control siRNA, vehicle + C/EBP-β siRNA and 1.0 mM MA + C/EBP-β siRNA after 24 h exposure. Bar graphs show the ratio of aggregated and monomeric JC-1 **(B)**. The percentage of apoptosis is presented as the mean ± SD **(D)**. ^∗^*p* < 0.05 vs. the saline vehicle-treated control group, ^#^*p* < 0.05 vs. the scrambled + MA group (one-way ANOVA).

To test the hypothesis that the C/EBP-β/lcn2 axis mediates MA-induced microglial apoptosis, we used siRNAs targeting lcn2 to silence its expression and then determined the bax, bcl2, cleaved caspase3, cleaved PARP and cytochrome c expression levels in MA-exposed BV-2 cells. The results of Western blotting showed that the expression levels of lcn2 and apoptosis-related proteins were increased by MA and decreased following lcn2 knockdown except the protein of C/EBP-β (Figures 7A–D). These results were further confirmed by flow cytometry (Figures 7E,F), the JC-1 assay (Figures 8A,B) and the TUNEL staining (Figures 8C,D). These results suggested that knockdown of lcn2 can effectively decrease MA-induced apoptosis in microglia.

### Silencing of C/EBP-β Expression Reduces MA-Induced Apoptosis *in vivo*

**FIGURE 9:**
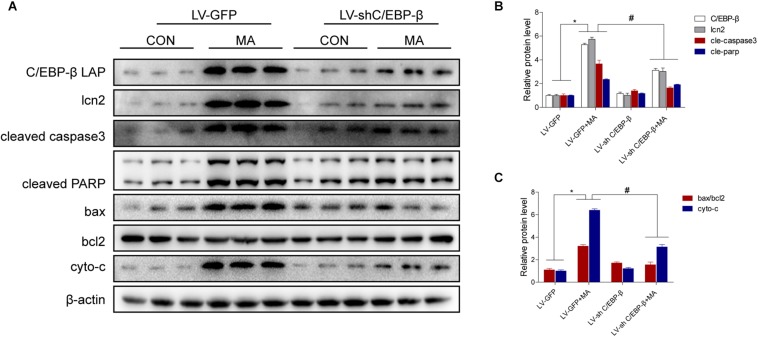
Silencing of C/EBP-β expression reduces MA-induced microglial apoptosis *in vivo*. LV-GFP and LV-shC/EBP-β lentiviruses were injected separately into the bilateral striatum of mice via a stereotaxic apparatus with saline or MA (15 mg/kg × 8 injections, at 12-h intervals, i.p.). Western blot and quantitative analyses were performed to determine the C/EBP-β, lcn2, cleaved caspase3 and PARP, ratio of bax/bcl2 and cytochrome c protein expression **(A–C)**. All data were expressed at least three independent replicates. β-actin was used as a loading control. The fold induction relative to that in vehicle-treated cells is shown. Data are presented as the means ± SD.^∗^*p* < 0.05 vs. the saline vehicle-treated LV-GFP group, ^#^*P* < 0.05 vs. the LV-GFP + MA group. Data were analyzed by one-way ANOVA.

To confirm the role of C/EBP-β in MA-induced microglial apoptosis *in vivo*, lentivirus vector (LV)-GFP and LV-shC/EBP-β were injected into the striatum of mice via a stereotaxic apparatus to silence the C/EBP-β expression in the striatal region (*n* = 5–8/group). After 4 days of stereotaxic injection, the mice were treated with saline or MA. Western blot analyses showed that MA-increased C/EBP-β protein expression in the LV-GFP group was significantly blocked by treatment with LV-shC/EBP-β in the mouse striatum. The expression levels of Lcn2, ratio of bax/bcl2, cytochrome c, cleaved caspase3, and cleaved PARP had similar effects (Figures 9A–C).

## Discussion

In this study, we provided evidence that MA, as a recreational drug, induced apoptosis in the microglial cell line BV-2 and in mouse brain tissues. Specifically, we found that MA-induced microglial apoptosis was due to the activation of mitochondrial apoptosis, and this pathway was mediated by the C/EBP-β-lcn2 axis.

Microglia are the resident macrophages of the CNS and represent 5 to 15% of adult brain cells, with densities varying between distinct brain regions (Allen and Lyons, 2018; Thion et al., 2018). Following stimulation with external stimuli, microglia undergo proliferation and morphological changes and play a key role in CNS homeostasis by mediating a protective response (Thion et al., 2018). Microglia are also phagocytic and phagocytize harmful substances and senescent cells, including abnormal neurons. However, prolonged exposure to toxic stimuli such as MA or secreted cytokines can lead to prominent neuronal dysfunction and impair microglial function, even leading to cell death, thereby resulting in worsened damage within the CNS. MA exposure contributes to microglial activation and secretion of inflammatory factors to promote neuroinflammation (Robson et al., 2013; Urrutia et al., 2013) and even induces microglial death through the Mir-143-BBC signaling pathway (Zhang et al., 2016). In the current study, we found that C/EBP-β mediated MA-induced microglial mitochondrial apoptosis.

Recent studies have also shown an upregulation of microglial C/EBP-β upon pro-inflammatory stimulation (Dasgupta et al., 2003; Ejarque-Ortiz et al., 2007, 2010). MA as a widely abused psychoactive drug, induced microglial activation and secretion of pro-inflammatory cytokines IL-1β, IL-6, and TNF-α (Wang et al., 2018). Pro-inflammatory cytokines IL-1β, IL-6, or TNF-α and LPS induced C/EBP-β upregulation in microglia through activation of the mitogen-activated protein kinases (MAPKs) signaling pathway (Ejarque-Ortiz et al., 2007). Furthermore, MA potentiates LPS stimulation of IL-1β production in microglia (Xu E. et al., 2018). We hypothesize that MA induced microglial reaction and secretion pro-inflammatory cytokines to involve in C/EBP-β expression. In addition, TLR4- HMGB1 involved in C/EBP-β expression in LPS-induced macrophage polarization (Gao et al., 2018). Our previous study has demonstrated that MA induced TLR4 upregulation in the microglia and astrocytes (Du et al., 2017). So the interaction of TLR4 with C/EBP-β in MA-induced microglia survival is a direction in the future research.

A number of studies have reported that C/EBP-β expressed in many tissues (Huber et al., 2012; Pulido-Salgado et al., 2015). C/EBP-β involved in neuronal death in our recent study (Xu X. et al., 2018) and others (Marshall et al., 2003; Grewal et al., 2009). LPS induces C/EBP-β upreglulation in astrocytes and microglia (Ejarque-Ortiz et al., 2007; Nakazato et al., 2015). The upregulation of microglial C/EBP-β in pro-inflammatory stimulation has been observed in the microglial cell lines BV-2 (Ejarque-Ortiz et al., 2010; Gresa-Arribas et al., 2010). In the present study, we found that C/EBP-β plays key roles in MA-induced microglial death, moreover, C/EBP-β is not only unique to microglia in the brain (Figure 1C). MA induced C/EBP-β upregulation in other non-identified cells possibly. Our previous study has found that MA induced C/EBP-β upregulation in neurons in the striatum (Xu X. et al., 2018). Further research are required to substantiate the C/EBP-β expression in diverse cell type after MA exposure. The interactions of microglia with neurons and other non-identified cells in MA-induced neurotoxicity are a direction in the future research. Knockdown of C/EBP-β with lentivirus can inhibit the apoptosis-related proteins bax, bcl2, cytochrome c, cleaved caspase3, and PARP and protect microglia from MA-induced toxicity *in vivo* and *in vitro*.

The function of lcn2 may be dependent on cell type because lcn2 can either stimulate or inhibit cell growth and differentiation in different cell types (Ferreira et al., 2015; Jha et al., 2015). Emerging evidences suggested that activated microglia *in vivo* may secrete lcn2 to facilitate morphological transformation and the secretion of cytokines (Lee et al., 2007, 2011; Naude et al., 2012). In microglia and astrocytes, the expression and secretion of lcn2 are increased under inflammatory conditions (Lee et al., 2009; Jang et al., 2013). Microglia- and astrocyte-derived lcn2 contributes to neuronal death during neurodegenerative conditions (Bi et al., 2013; Tong et al., 2013). Series reports indicated that lcn2 is involved in mitochondrial apoptosis and mediates cell apoptosis (Devireddy et al., 2005; El Karoui et al., 2016). Lee et al. (2007) reported that lcn2 makes microglia more vulnerable to apoptotic stimuli. We found that lcn2 is involved in MA-induced microglial apoptosis. In addition, C/EBP-β are implicated in the transcriptional control of lcn2 (Jha et al., 2015). Positions for putative binding sites of transcription factor of C/EBP-β was −190 and −170 nt in the lcn2 promoter were measured by the luciferase reporter assay (Fujino et al., 2006). Both the NF-κB and C/EBP-β sites in the Lcn2 promoter are important for IL-17-mediated transcription (Shen et al., 2006). In this study, C/EBP-β was shown to be a transcription factor involved in lcn2 after MA-induced microglial apoptosis.

BV-2 is a certainly popular cell line to study microgla, Some researchers study pathophysiological functions of microglia using BV-2 cells in numbers of studies, including MA exposure (Dasgupta et al., 2003; Guo et al., 2015; Zhang et al., 2016). However, BV-2 cells do not always reproduce well the phenotype of primary microglia or *in vivo* microglia (Butovsky et al., 2014). This is a limitation of our research. Further research are required to substantiate the present findings using C/EBP-β knockout animal models.

**FIGURE 10:**
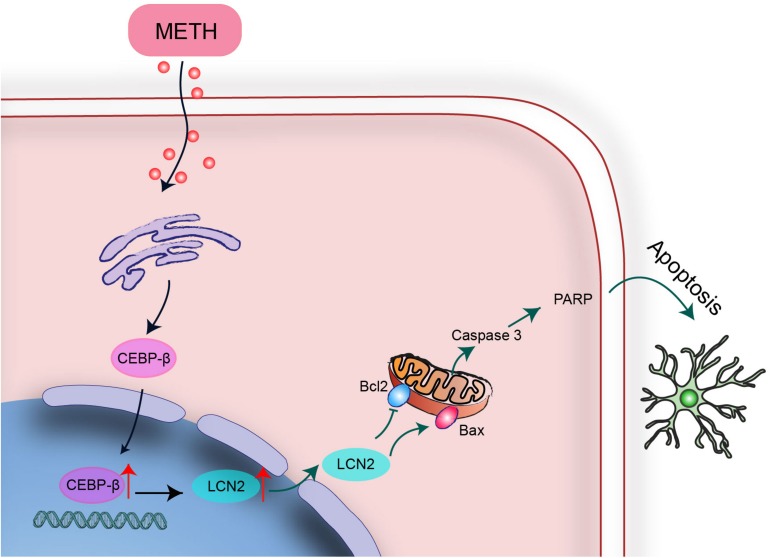
A schematic depicting the role of the C/EBP-β-lcn2 signaling pathway in MA-induced microglial apoptosis. A schematic depicting the role of the C/EBP-β/lcn2 signaling pathway in MA-induced microglial apoptosis via a mitochondrial-dependent apoptosis pathway. Briefly, C/EBP-β expression is induced after MA exposure, which upregulates the expression level of lcn2 by transactivating its promoter. Then, lcn2 triggers apoptosis through the mitochondrial pathway.

In summary, C/EBP-β upregulation might be a potential pathogenic mechanism leading to further disruption of the striatum induced by MA neurotoxicity. C/EBP-β is involved in MA-induced microglial apoptosis *in vivo* and *in vitro*. lcn2 is a downstream target of C/EBP-β-mediated MA-induced apoptosis. Knockdown of C/EBP-β decreased lcn2 expression to protect microglia from MA-induced apoptosis (Figure 10). These results suggested that MA-induced microglial apoptosis could be partly reversed by knocking down C/EBP-β. These findings provide a new insight regarding the specific contributions of C/EBP-β-lcn2 to microglial survival in the context of MA abuse.

## Data Availability

The authors declare that all data supporting the findings of this study are available within the article.

## Author Contributions

XC and XY conducted all the experiments with the help of JL, DQ, CC, and XZ. XC and HW designed the experiments. XC, HW, and XY analyzed and interpreted the results. HW and XC wrote the manuscript with the help of XY, JL, and XZ. All authors approved the final manuscript.

## Conflict of Interest Statement

The authors declare that the research was conducted in the absence of any commercial or financial relationships that could be construed as a potential conflict of interest.

## Abbreviations

AICD: activation-induced cell death
BSA: bovine serum albumin
C/EBP-β: CCAAT-enhancer binding protein
CNS: central nervous system
LSD: least significant difference
LPS: lipopolysaccharide
MA: methamphetamine
SD: standard deviation
siRNA: small interfering RNA
SMU: Southern Medical University.
