# Supplementary material for: Role of C/EBP-β in Methamphetamine-Mediated Microglial Apoptosis
Source: Front Cell Neurosci. 2019 Aug 21;13:366. doi: 10.3389/fncel.2019.00366 (PMC6712175; doi:10.3389/fncel.2019.00366)
Supplement: Supplementary file 1 [file Data_Sheet_1.pdf]

Supplementary materials

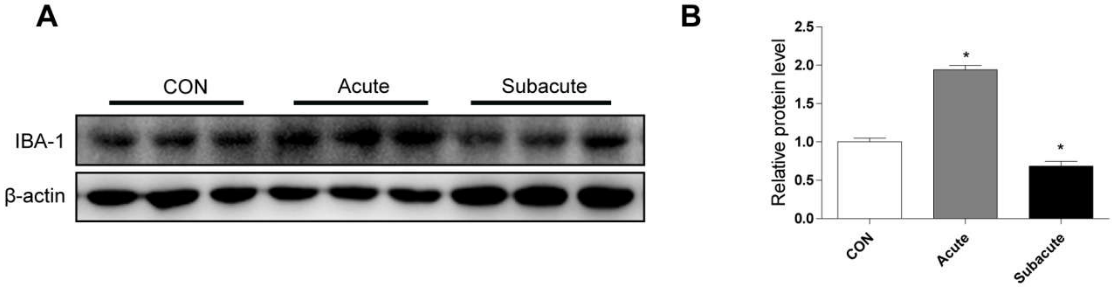

Supplement FIG1 MA exposure induces IBA-1 expression *in vivo*. Representative Western blots (A) and quantitative analyses(B) of IBA-1 in the corpus striatum tissues of mice from the control group, acute group and subacute group. N=5-8 animals/group. Fold induction relativeto the vehicle-treated group(*p<0.05 vs. the vehicle-treated group using one-way ANOVA). β-actin was used as a loading control. All data are presented as the mean ± SD.

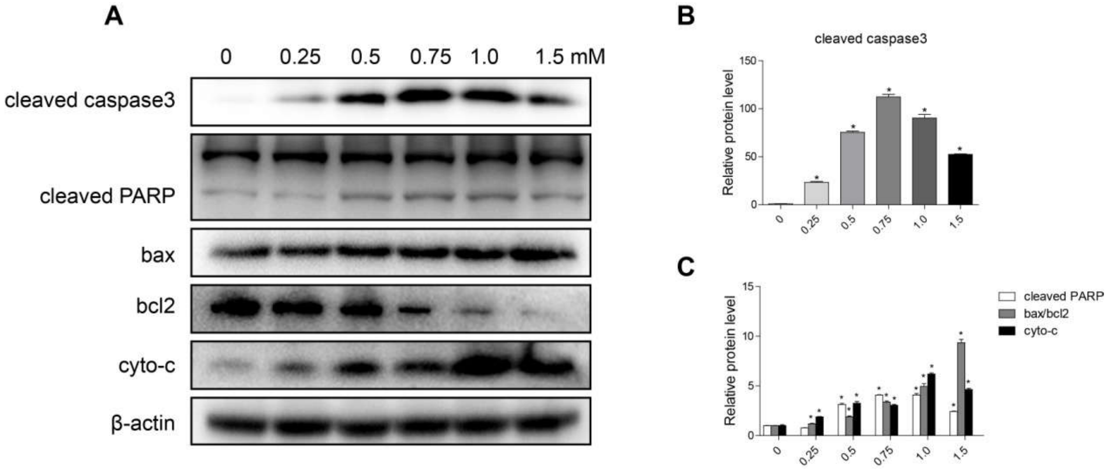

Supplement FIG 2 MA increases apoptosis in a dose dependent manner. Microglial line BV 2 cells were treated with 0.25–1.5 mM MA for 24 h (A-C). The protein expression levels of the cleaved caspase3 and cleaved PARP, ratio of bax/bcl2 and cytochrome c were determined with Western blot and quantitative analyses. β-actin was used as a loading control. The fold induction relative to that in vehicle-treated cells is shown. Data are presented as the means ± SD. *P <0.05 vs. the saline vehicle-treated control group. Data were analyzed by one-way ANOVA.
